# Hazard, Distribution and Exposure of Particulate Pollution from Indoor and Outdoor Environments

**DOI:** 10.3390/toxics11090772

**Published:** 2023-09-12

**Authors:** Maurizio Gualtieri, Marie Carriere, Paride Mantecca

**Affiliations:** 1 Department of Earth and Environmental Sciences, Research Centre POLARIS, University of Milano-Bicocca, Piazza della Scienza 1, 20126 Milan, Italy; paride.mantecca@unimib.it; 2 University Grenoble-Alpes, CEA, CNRS, IRIG, SyMMES, CIBEST, 38000 Grenoble, France; marie.carriere@cea.fr

Air is an essential natural resource for life. Clean air, in indoor and outdoor environments, is in fact a primary environmental factor for the wellbeing of humans. In the past years, the effects of poor air quality have been extensively studied and their impact on the quality of life of human population is a matter of fact. Negative effects of airborne pollution are cardiovascular diseases [1,2,3], respiratory diseases [4,5,6], increased general mortality and other emerging symptoms, among which also neuroinflammatory and neurodegenerative diseases [7,8,9].

Air pollution is a mixture of different chemical (organic and inorganic) and physical (particles or gases) species (of natural or anthropic origin), and differences exist considering indoor and outdoor environments. Airborne particles are grouped among different classes according to their aerodynamic diameter. PM10 comprises all the particles with a mean aerodynamic diameter lower than 10 microns, PM2.5 the particles with a mean aerodynamic diameter lower than 2.5 microns, PM1 all the particles with mean aerodynamic diameter lower than one micron. In the recent years, the ultrafine particles (UFPs), which represents the particles with aerodynamic diameter lower than 100 nm, become of primary interest for understanding the impacts of airborne pollution on health. UFPs typically generate during combustion processes (of natural or anthropogenic origin) and given their tiny dimensions may deposit deep in the lung and translocate to other organs. Furthermore, the continuous development during the last two decades of engineered nanoparticles (NPs), that may be defined as particles with a least one dimension lower than 100 nm, constitute an additional burden to air pollution by these sub-micrometric particles. Their presence in outdoor and indoor air may determine a potential additional exposure for the general population but also specific sub-groups, like workers involved in nanotech manufacturing and final users of nano-enabled products (NEPs), who may be even more at risk of exposure. Such intended production and use of NEPs may also determine the exposure through other routes than inhalation, such as ingestion or dermal contact, thus increasing the potential overall risk.

The understanding of potential health risks of UFPs and NPs and specifically associated pollutants is a complex field of research that requires integrative and multidisciplinary approaches. Three complementary pillars may be defined for these approaches. The first is the hazard evaluation by means of in vitro and in vivo toxicological studies, considering the more appropriate models for target tissues or organs (e.g., lungs, brain, skin, liver, or intestine). Secondly, the evaluation of the actual human exposure by means of environmental campaigns (at relevant outdoor or indoor environments), eventually complemented by laboratory or modelling approaches and finally data analysis and merging to provide the more relevant information for human risk assessment. The final goal is to provide information to reduce and/or prevent the diseases arising from the unwanted exposure to particulate pollution. 

These approaches are usually developed and applied *a posteriori*, i.e., when the pollution is already affecting an environmental compartment. To overcome this, novel frameworks are developing. When considering engineered NPs, the Safe and Sustainable by Design (SSbD) approach [10] is a guide to avoid unwanted risks during the production or use of novel particles. Among the SSbD framework aims, the reduction of hazards toward humans and the whole environment plays a key role. In SSbD approaches the environment is considered starting from the very first steps of development of the new materials and therefore difference target species, beside humans, are considered. The framework consists in a recursive approach that requires the testing of the new material after every modification or during every subsequent step of its life cycle (production, use, reuse, and disposal). In this context, also the Adverse Outcome Pathway approach [11] is a relevant reference to plan toxicological experiments, targeting from the beginning significant human diseases and therefore providing proofs of the potential link between exposure to a pollutant and onset of a disease. 

The complexity of the procedures, required to obtain data for a proper risk assessment or to develop new NPs or NEP under the SSbD framework, opens many challenges for researchers engaged from different disciplines, like chemistry, biology, toxicology, physics, environmental sciences, engineering and so on. 

Accordingly, this special issue, “Nano and Ultrafine Particle Toxicology and Exposure Assessment”, represents a collection of papers reporting on the main achievements obtained by different scientific communities involved in the hazard characterization of particles and associated chemicals, representative of both indoor and outdoor environments. The effects of these pollutants are here tested on cells representative of target organs in humans (lungs, intestine, or brain) but also on organisms that are representative of specific environmental compartments, i.e., fresh waters. Finally, the potential exposure of humans to particles is studied starting from data collected by environmental monitoring campaigns (at production or outdoor sites), with the final aim of providing robust exposure data for risk assessment frameworks.

A better understanding of population exposure concentration and doses, of the hazard of particulates of novel development or originating from canonical human activities are essential for the development of novel regulatory actions or policies aiming at reducing risk. In a One Health approach, human health is only a part of the complexity of interaction with all the other species living on Earth, and future research should consider implementing comparative studies considering also other species beside humans. 

We would like to thank all the authors that have considered and submitted their original manuscripts to this special issue. A special acknowledgment to the reviewers that used their time to review the submitted manuscripts providing useful reports for their improvement. We would like to thank also the Toxics editors for inviting us as guest editors and Selena Li for her help and support. 
